# Concordance of Australian state and territory government guidelines for classifying the healthiness of foods in public settings

**DOI:** 10.1017/S1368980025000059

**Published:** 2025-02-03

**Authors:** Bettina Backman, Meg Adam, Jasmine Chan, Josephine Marshall, Emalie Rosewarne, Gary Sacks, Adrian J. Cameron, Miranda R. Blake

**Affiliations:** 1 Deakin University, Global Centre for Preventive Health and Nutrition (GLOBE), Institute for Health Transformation, Geelong, VIC 3220, Australia; 2 Deakin University, Institute for Physical Activity and Nutrition, Geelong, VIC 3220, Australia; 3 The George Institute for Global Health, The University of New South Wales, Sydney, NSW 2052, Australia

**Keywords:** Food classification, Food policy, Australia, Nutrition standards, Public settings

## Abstract

**Objective:**

To investigate the concordance between Australian government guidelines for classifying the healthiness of foods across various public settings.

**Design:**

Commonly available products in Australian food service settings across eight food categories were classified according to each of the seventeen Australian state and territory food classification guidelines applying to public schools, workplaces and healthcare settings. Product nutrition information was retrieved from online sources. The level of concordance between each pair of guidelines was determined by the proportion of products rated at the same level of healthiness.

**Setting:**

Australia.

**Participants:**

No human participants.

**Results:**

Approximately half (56 %) of the 967 food and drink products assessed were classified as the same level of healthiness across all fifteen ‘traffic light’-based systems. Within each setting type (e.g. schools), pairwise concordance in product classifications between guidelines ranged from 74 % to 100 %. ‘Vegetables’ (100 %) and ‘sweet snacks and desserts’ (78 %) had the highest concordance across guidelines, while ‘cold ready-to-eat foods’ (0 %) and ‘savoury snacks’ (23 %) had the lowest concordance. In addition to differences in classification criteria, discrepancies between guidelines arose from different approaches to grouping of products. The largest proportion of discrepancies (58 %) were attributed to whether products were classified as ‘Red’ (least healthy) or ‘Amber’ (moderately healthy).

**Conclusions:**

The results indicate only moderate concordance between all guidelines. National coordination to create evidence-based consistency between guidelines would help provide clarity for food businesses, which are often national, on how to better support community health through product development and reformulation.

Food environments across the globe are dominated by the promotion of unhealthy foods that are high in sugar, salt and/or saturated fat^(1)^. Evidence shows that even government-owned and operated settings, such as schools, healthcare settings and sports and recreation centres, frequently offer and promote foods and drinks that have low nutritional quality both globally^(2)^ and in Australia^(3–5)^. The WHO emphasises the importance of establishing government-led food and nutrition policies to promote healthier food choices in government-owned settings^(6)^. For such policies, foods and drinks often need to be classified using food-based and/or nutrient-based systems (including nutrient profiling)^(2,7)^. Consistent implementation and monitoring of nutrition policy-driven healthy changes in food outlets can be helpful to increase the availability of healthy options and to guide evidence-based practice^(2)^. Despite this, setting-specific nutrition policies often vary between and within countries in their application (e.g. mandatory or voluntary), the level of monitoring of implementation and the food classification criteria and guidelines used^(2,7,8)^. The impacts of different approaches to food classification within countries can include duplication of work across jurisdictions, barriers to food reformulation initiatives by the food industry (e.g. unclarities around which classification criteria to use), vast differences in healthiness classifications^(9)^ and confusion among retailers who are expected to implement healthy changes in their product offerings^(10,11)^ While varying approaches may sometimes be needed for different purposes (e.g. restrictions on unhealthy food marketing *v*. food provision)^(12,13)^, differences in classification systems developed for the same purpose (e.g. food provision in schools) across jurisdictions within the same country may lead to confusion and hinder policy implementation.

In Australia, government-owned settings have been identified by the state and federal governments as high priority settings to promote healthier diets across the population and to children specifically^(14–17)^. Each state and territory government has produced its own setting-specific food classification guidelines to guide food service providers as to what constitutes a healthy food offering within these settings^(18)^. While a national approach for school canteens has been provided through the ‘Healthy School Canteen Guidelines’^(19)^, most states have opted to develop their own guidelines^(18)^. Often, nutritional criteria and food-based guidance are used to classify foods using traffic light colours of ‘Green’ (healthiest), ‘Amber’ (moderately healthy) and ‘Red’ (least healthy) with a recommended percentage of how much of the menu should be made up of products of each classification. Guidance on in-store marketing practices (e.g. pricing, promotion and placement) also uses this traffic light categorisation of food healthiness^(18)^. Uniquely within Australia, the state of New South Wales has adopted a system that utilises the national voluntary health star rating (HSR) system to classify products into ‘Everyday’ and ‘Occasional’ categories^(16,18,20)^. The HSR system gives products 0·5 to 5 ‘stars’ based on their nutritional profile, with a higher HSR indicating that the product is a healthier option compared with other similar products in that category^(21)^. While all Australian state and territory food classification guidelines are underpinned by the Australian Dietary Guidelines (ADG)^(22)^, previous research has found high variability in how nutrient criteria are used to categorise food products across twenty-four nutrition policies applying to schools, healthcare settings, workplaces and other government-owned settings^(18)^. Similar research has also found differences between recommended food provision guidelines for early childhood education settings across Australian jurisdictions^(23)^. Neither of these studies investigated the level of concordance between the guidelines (i.e. the percentage of products similarly classified by different guidelines). Evidence gaps therefore remain in understanding what the implications of varying Australian state government approaches to food classification are for real-world food classification outcomes (i.e. what percentage of products are differently classified).

This study aimed to investigate the concordance between Australian state and territory government guidelines for classifying the healthiness of foods across various government-owned settings and to identify the main factors causing differences in food classification outcomes.

## Methods

The study involved four steps: (1) updating a database of national, state and territory food classification guidelines; (2) collecting a sample of common food products in government-owned food service settings; (3) classification of food and drink products according to each classification system and (4) assessing the concordance between guidelines.

### Step 1: Updating a database of national, state and territory food classification guidelines

A database of food classification criteria from national, state and territory guidelines on food provision in government-owned foodservice settings including schools, hospitals, workplaces, sport and recreation facilities was created by Rosewarne et al. in 2019^(18)^. This was updated for the current study by conducting a Google search for new editions of relevant guidelines released between 2019 and May 2023, following the process described in the original study^(18)^. Guidelines that were not publicly available from online sources (e.g. state government websites) between May and August 2023 were excluded. Data extracted from each guideline for all food and drink categories included: nutrition information (energy, fat, saturated fat, Na, total sugar and fibre per 100 g or per serve), serving size criteria, HSR (NSW guidelines) and any additional criteria affecting the product classification (e.g. fruit juice content in fruit drinks). How the food classification applied to restrictions for food availability, fundraising, catering, advertising, marketing, pricing and display was also documented.

### Step 2: Collecting a sample of common food products in government-owned food service settings

To illustrate the implications of differences in setting-specific guidelines for food classification, nutrition information was compiled for a sample of foods and drinks between November 2021 and May 2023. The selection of food and drink categories of interest was informed by prior research by Rosewarne et al. (2019)^(18)^, which identified prevalent food categories included in the Australian state and territory government food classification guidelines: ‘Cakes/sweet tarts/pastries’, ‘Savoury snacks’ (e.g. savoury biscuits and crisps), ‘Processed meat’ (cold luncheon and cured meats), ‘Meat products’ (e.g. crumbed and coated meat and frankfurters), ‘Hot food items’ (e.g. savoury pastries, pizza and instant noodles), ‘Pre-prepared meals’, ‘Ice-creams and dairy desserts’, ‘Sweet snacks’ (e.g. muesli bars and sweet biscuits), ‘Pre-packaged dairy drinks’ and ‘Non-dairy drinks’. Additionally, those product categories contributing the most energy from discretionary foods according to the Australian Health Survey (2011–2012) were also included^(24)^, namely ‘Cakes, muffins scones and cake-type desserts’, ‘Confectionery and cereal/nut/fruit/seed bars’, ‘Pastries’, ‘Sweet biscuits’ and ‘Savoury biscuits’ and ‘Soft drinks and flavoured mineral waters’. Alcoholic beverages were excluded from this study as they are not served in most government-owned food service settings.

The sample of food products included in this study were from two different sources: (i) top-selling packaged food and drink brands and (ii) food products from real-world community food retail outlets.

#### Food products from top-selling packaged foods brands

A range of food products in each of the target categories was selected from top selling brands according to sales value reported by Euromonitor in Australia in 2020^(25)^. Sixty products were included per product category of interest (e.g. ‘cakes/sweet tarts/pastries’). Starting with the three top selling brands in each category, up to twenty products per brand were included by systematically selecting every second product listed on the manufacturer’s website. If the nutrition information for the top three highest-selling brands was not available online, or these brands included less than sixty eligible products, then the next highest-selling brand was also included until sixty products per category was reached. Supermarket-owned brands were excluded, as it was assumed that these are not typically available in food service settings.

#### Food products from real-world community food retail outlets

A convenience sample of seven food retail outlets in community-based sporting settings (e.g. public swimming pools and sports complexes) in metropolitan (*n* 6) and regional (*n* 1)^(26)^ Victoria were recruited via existing networks to obtain a database of products available in real-world settings. Outlets were chosen based on the range of different product types available, including products prepared onsite. A record of all food and drink products available for purchase in each outlet was collected. The lead author and university nutrition placement students obtained copies of the menus and conducted photographic audits of food displays and products between February and June 2022. Initially, all real-world products from the first three outlets were combined with the sample of top-selling products and duplicates were removed. Additional ‘Cold ready-to-eat meal’ products were then purposively selected from four additional outlets to ensure comprehensive representation of this category (at least thirty products).

#### Combining real-world and top-selling product samples

Real-world and top-selling product samples were combined to create product categories and subcategories, which were used to compare the concordance between guidelines and to allow more detailed analysis of discrepancies between guidelines. This was done by grouping together products subject to similar classification criteria (e.g. total energy, Na and saturated fat content were commonly used to classify ‘savoury snacks’). For example, uncoated and coated seafood products were categorised separately from meat products as uncoated seafood products were often classified based on ingredients (e.g. canned tuna in water *v*. canned tuna in oil), while coated seafood products were subject to nutrient criteria that differed from the criteria for meat products in some guidelines.

#### Data extraction

The following information was extracted for each product included in the sample (where available): nutrition information (energy, protein, carbohydrate, sugar, fat, saturated fat, sodium and fibre content), ingredients list and serving size. The data were extracted from food packaging (product photos), recipes or manufacturer, supplier or retailer websites. Nutrition information for products prepared by food outlets was estimated using the Australian Food and Nutrient Database 2011–2013^(27)^ by finding the closest match in the database to the product prepared onsite. For example, for a sandwich made with ham, the corresponding product in Australian Food and Nutrient Database would be ‘Sandwich or roll, filled with ham’.

### Step 3: Classification of food and drink products according to each classification system

#### Classification according to each guideline

Packaged products were classified according to each guideline based on the data extracted in Step 2, and products prepared onsite were classified using the available recipes. Manufacturer serving size was used to determine product classification where serving size limits applied. FoodChecker, a tool designed for assessing the healthiness of foods according to South Australian, West Australian, Victorian and Queensland guidelines, was used to classify products prepared onsite (e.g. sandwiches and salads) for Victoria and Queensland^(28)^. FoodChecker was not available for South Australia, West Australia or other jurisdictions at the time the products were classified.

Each product was classified as ‘Green’ (healthiest), ‘Amber’ (moderately healthy) or ‘Red’ (least healthy) against all publicly available state guidelines applying to schools, healthcare settings and workplaces. Beyond these commonly used traffic light categories, the Victorian school nutrition guideline includes a ‘Black’ category, and the South Australian school nutrition guideline includes a ‘Red 2’ category, both aimed at identifying products that are not permissible to be sold in schools. Products falling into ‘Black’ and ‘Red 2’ categories were first categorised separately and later grouped together with ‘Red’ category to allow comparison with other traffic light-based guidelines. In NSW, where the traffic light-based system is not used, products were classified as ‘Everyday’ or ‘Occasional’ foods instead. Products that did not meet the criteria for the NSW ‘Occasional’ category were classified as ‘Banned’, as these products are not permitted in these settings^(16,20)^.

#### Classification according to national schemes

Products were classified using nationally recognised schemes to assess how well the state-based guidelines align with different national schemes in categorising ‘healthy’ and ‘unhealthy’ foods. Products were rated using the HSR system^(21)^ also to allow comparison between the traffic light-based systems and the New South Wales HSR-based system. HSR was sourced from the manufacturer’s website if available or otherwise calculated using the HSR calculator with the updated 2020 algorithm^(21)^ and relevant guidelines accompanying the calculator^(29)^. Additionally, products were classified according to the ADG^(22)^ as ‘discretionary’ (not necessary for a healthy diet) or ‘core’ (based on five core food groups relevant to a healthy diet: fruits; vegetables; grains; lean meats and alternatives and dairy and alternatives), as well as against the Council of Australian Government’s (COAG) ‘National interim guide to reduce children’s exposure to unhealthy food and drink promotion’^(30)^. The COAG guide provides minimum standards for jurisdictions to develop state-based guidelines to reduce children’s exposure to unhealthy food marketing^(30)^.

#### Coding process

All product classifications were completed by researchers (BB, JC, JM and MRB) and university nutrition placement students, using the relevant product information documented in Step 2 and comparing it with the classification criteria for each scheme. For traffic light codes, 500 of the products were double coded by two members of the research team (BB, JC), and classification outcomes for each product category were reviewed during the data analysis. Any disagreements were resolved by the second coder who reviewed the original guideline documents to re-classify the products where required. Out of 9265 individual classifications reviewed (500 products across 17 classification systems), only 565 required changes, indicating a high interrater reliability of 94 % among coders.

### Step 4: Assessing the concordance between the guidelines

#### Concordance between traffic light-based systems

The proportion of products scoring the same traffic light classification across guidelines was determined. For each product, concordance between each guideline applying to the same setting (e.g. schools) was indicated if guidelines placed the same products in the same ‘healthiness categories’ (‘Green’, ‘Amber’ and ‘Red’) as each other. Level of agreement between each pair of guidelines was also assessed using a pairwise weighted Cohen’s κ statistic, which is used with ordinal variables and considers the possibility of the agreement occurring by chance. Weighted Cohen’s κ was interpreted as follows: κw = 0·81–1 was considered ‘almost perfect’ concordance, κw = 0·61–0·80 was considered ‘substantial’, κw = 0·41–0·60 was considered ‘moderate’ and κw = 0·21–0·40 was considered ‘fair’^(31)^.

#### Concordance between traffic light-based and Health Star Rating-based systems

Mean HSR across traffic light system categories and ‘Occasional’/’Everyday’ categories (for NSW) for included products was also determined to compare traffic light-based systems and the New South Wales HSR-based system. A series of one-way ANOVA were conducted to compare the mean HSR of products falling into similar healthiness categories across the guidelines. Post hoc analysis using Tukey’s honestly significant difference test was also conducted to identify, which pairwise comparisons between guidelines showed different mean HSR within healthiness categories where significant results were found.

## Results

Seventeen government-led state and territory level setting-specific food classification guidelines across Australian jurisdictions were included for comparison, including eight guidelines for schools, seven guidelines for healthcare facilities and two for workplace. Appendix I summarises the food classification guidelines included in this study. Ten^(16,17,32–39)^ out of seventeen guidelines were updated since the database by Rosewarne et al.^(18)^ was created in 2019. Seven of the eight jurisdictions continued to use a ‘traffic light’-based system to classify food and drink products. Only New South Wales did not use this approach, opting instead to continue utilising the HSR to classify products into ‘Everyday’ and ‘Occasional’ categories (Appendix I). Differences in classification criteria across jurisdictions remained evident. The minimum criteria that products must meet to avoid being classified as least healthy (‘Red’) or being prohibited in schools and healthcare facilities across all jurisdictions are summarised in Appendix II. Guidelines had differing approaches relating to the proportion of products falling into each healthiness category permitted in food outlets and the way healthiness categories are used to guide the in-store marketing (e.g. placement) of products (Appendix I). For example, most school nutrition policies did not allow any ‘Red’ products in school canteens, while some guidelines applying to workplaces and healthcare settings allowed these products to constitute up to 20 % of the product offering.

### Product classifications

A total of 1001 products from top selling brands and real-world food outlets were included in the product list. Thirty-four products were excluded from the comparison: twenty were duplicates, twelve products were ‘recipe bases’ or primarily used as ingredients and nutrition information could not be retrieved for two products. A total of 967 products were included for comparison, including 769 food and 198 drink products. Products from real-world food outlets accounted for 18 % (*n* 173) of the sample. Products were categorised into eight major product categories (e.g. non-dairy drinks) and twenty-four subcategories (e.g. soft drinks). Most products (*n* 653, 68 %) were classified as ‘discretionary’ based on the ADG^(22)^ and ‘unhealthy/not recommended for promotion’ (*n* 754, 78 %) based on the COAG guidelines^(30)^. More than one in three products (*n* 354) were ‘sweet snacks and desserts’ (e.g. cakes, slices and sweet pastries). Appendix III summarises the categories, subcategories and frequency of products included in the study.

**Figure 1.**
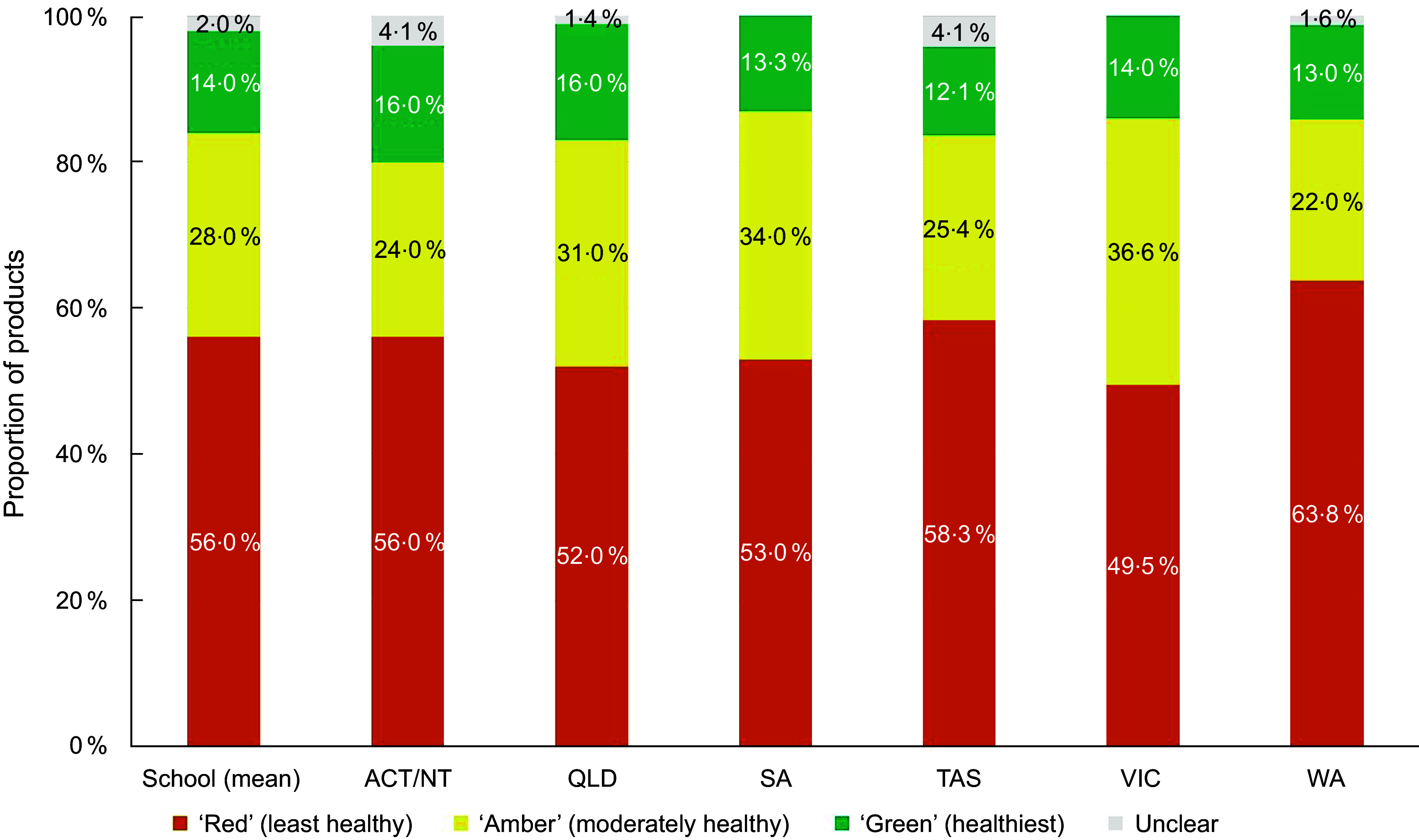
Proportion of products falling into each healthiness category across guidelines applying to schools in an assessment of 17 Australian setting-specific nutrition guidelines in 2021–2023.

**Figure 2.**
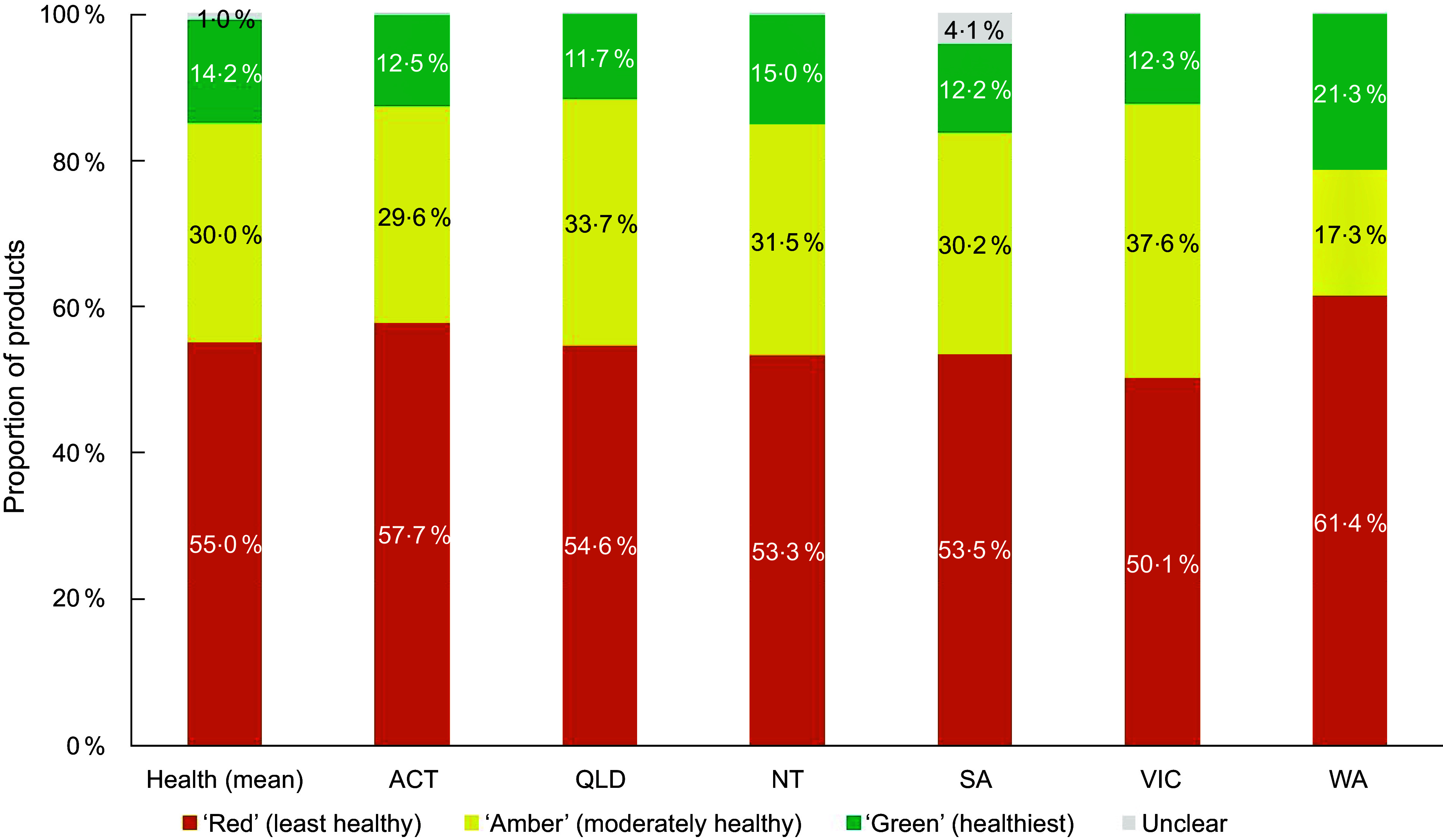
Proportion of products falling into each healthiness category across guidelines applying to healthcare facilities in an assessment of 17 Australian setting-specific nutrition guidelines in 2021–2023.

**Figure 3.**
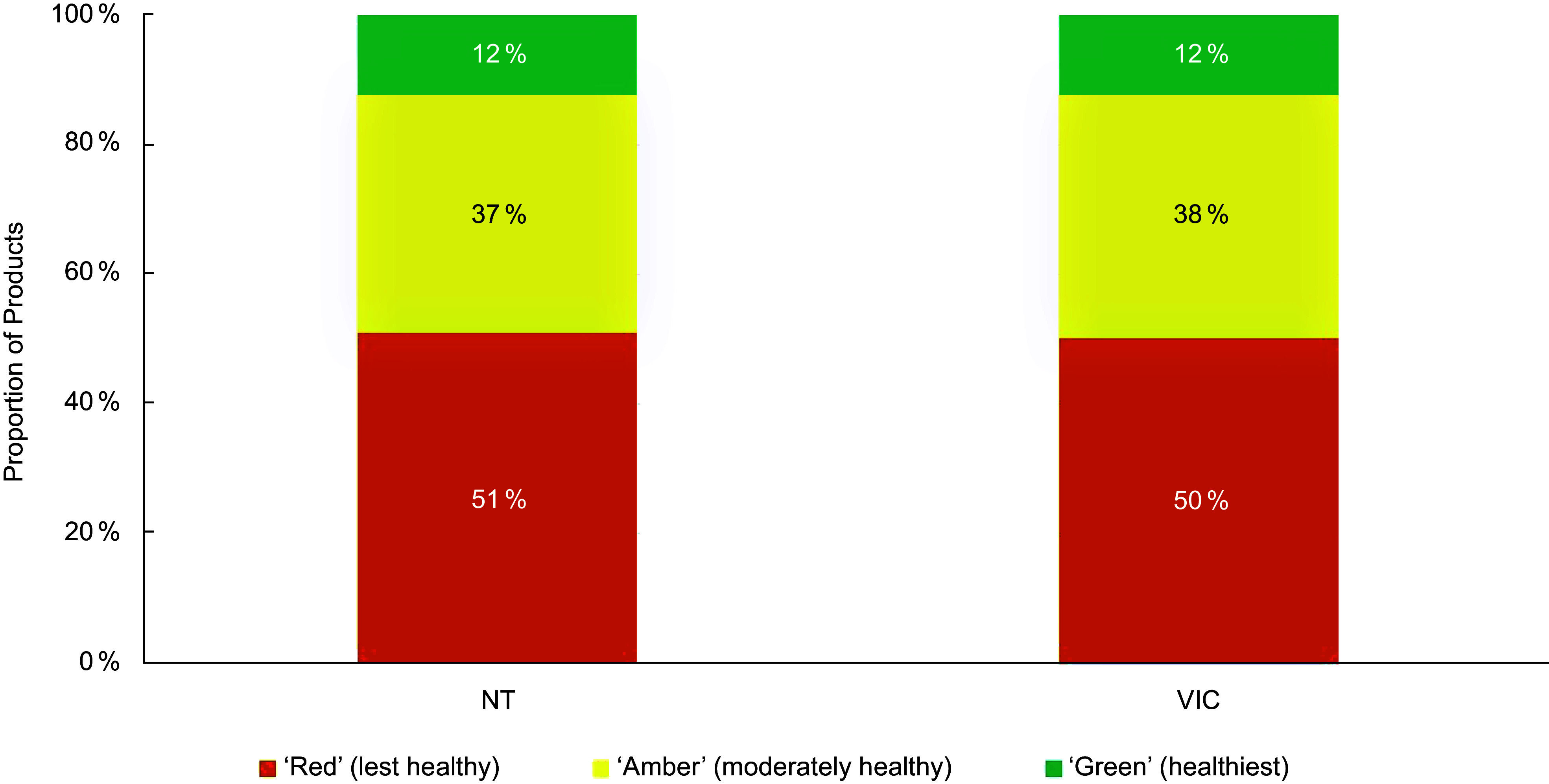
Proportion of products falling into each healthiness category across guidelines applying to workplaces in an assessment of 17 Australian setting-specific nutrition guidelines in 2021–2023.

When considering product classifications using traffic-light based guidelines only, most products were classified in the least healthy ‘Red’ category (mean across guidelines: 55 %; range between guidelines: 49–64 %). On average, 30 % of the products were classified as ‘Amber’ (range 17–39 %) and 14 % were classified in the healthiest ‘Green’ category (range 12–21 %). Figures 1–3 compare the proportion of assessed products falling into each classification category by guideline setting. The proportion of products falling into ‘Amber’ classification category varied the most between guidelines for schools (range 22–37 %; Figure 1) and health facilities (range 17–38 %; Figure 2), with West Australian (WA) guidelines for health facilities placing least products (17 %) and Victoria placing most products into this category across all setting types (37–38 %). Of all policy setting guidelines, WA guidelines categorised the highest number of products as ‘Red’ (64 % for schools, 61 % for healthcare). However, the WA health facilities guidelines categorised more products as ‘Green’ (21 %) compared to other schemes (Figure 3). The proportion of products categorised as ‘Green’ was more consistent across guidelines for schools compared to guidelines for health facilities (Figures 1–2).

For the NSW health facilities guidelines, 25 % (*n* 246) fell into the healthiest ‘Everyday’ and 39 % (*n* 381) into the moderately healthy ‘Occasional’ category. For NSW school guidelines, 24 % (*n* 236) of products were classified as ‘Everyday’ and 17 % (*n* 169) as ‘Occasional’. The remaining products (58 % for schools, 33 % for healthcare) were ‘Banned’ according to this classification system. A small number of products from the real-world sample (‘Cold ready-to-eat meals’) were coded as ‘unclear’ due to ambiguous or missing classification criteria for these products in some guidelines (e.g. lack of clear criteria for products prepared onsite). For example, some guidelines (*n* 4) included classification criteria for ingredients used in foods that can be prepared onsite, such as salads and sandwiches (‘cold meals’) but did not provide unambiguous instructions on how to classify the whole product when pre-prepared. Thus, such products could not be classified for the purpose of this study using the guidelines that lacked clear instructions for classifying the whole product.

### Concordance between guidelines

#### Concordance between traffic light-based systems

Overall, 56 % of products assessed were classified in the same healthiness categories across all guidelines using traffic light system applying to all three setting types. The concordance across all guidelines for schools was higher (71 %, *n* 8 guidelines) compared to all guidelines for healthcare facilities (66 % concordance; *n* 7 guidelines). Workplace nutrition guidelines had the highest concordance across setting types, with 87 % of products falling into the same healthiness categories; however, this high concordance was likely due to a smaller number of workplace guidelines available (*n* 2).

Pairwise concordance between guidelines using traffic light systems for each setting type was ‘moderate’ to ‘almost perfect’, with the proportion of products classified at the same level of healthiness ranging from 74 % to 100 % (mean 85 %, Cohen’s κ: 0·56–1; Table 1). Two of the school nutrition policies (ACT and NT) used the same classification guidelines resulting in perfect concordance in product classification between these jurisdictions. As for policies using different classification guidelines, the highest pairwise concordance was between SA and NT guidelines for healthcare facilities (96 %; κw 0·92), while the lowest concordance was between SA and WA guidelines for healthcare facilities (74 %; κw 0·56). On average, 58 % of the discrepancies between guidelines were attributed to whether a product was classified as ‘Red’ or ‘Amber’, 25 % were attributed to whether a product was classified as ‘Amber’ or ‘Green’, 7 % were attributed to whether a product was classified as ‘Green’ or ‘Red’, and the remaining 10 % were related to unclear classification outcomes. Products that were classified as ‘Green’ in one guideline and ‘Red’ in another included flavoured water (without intense sweeteners), uncoated seafood products and convenience meals.

**Table 1.** Cohen’s κ statistics and percentage agreement between guidelines in each jurisdiction using traffic light-based systems by setting type in assessment of 17 Australian setting-specific nutrition guidelines in 2021–2023

	ACT		QLD		NT		SA		TAS		VIC	
	κw	%	κw	%	κw	%	κw	%	κw	%	κw	%
Schools												
ACT												
QLD	0·77	85·2										
NT	*1* ^*^	*100* ^*^	0·77	85·2								
SA	0·73	83·6	0·81	88·4	0·73	83·6						
TAS	0·89	90·1	0·72	82·5	0·89	90·1	0·74	84·9				
VIC	0·69	81·3	0·77	86·1	0·69	81·3	0·80	87·8	0·68	80·8		
WA	0·73	83·7	0·70	82·0	0·73	83·7	0·70	80·7	0·73	84·1	0·61	77·4
Healthcare												
ACT									.	.		
QLD	0·81	89·9							.	.		
NT	0·73	84·2	0·75	85·5					.	.		
SA	0·68	81·5	0·72	83·5	0·92	95·7			.	.		
VIC	0·78	87·1	0·82	89·7	0·74	84·7	0·76	82·1	.	.		
WA	0·68	80·9	0·64	78·7	0·60	75·5	0·56	73·7	.	.	0·58	74·8
Workplaces												
NT	.	.	.	.			.	.	.	.		
VIC	.	.	.	.	0·79	87·4	.	.	.	.		

Cells with ‘.’ indicate the absence of guidelines for that setting within the jurisdiction.

Green cells = ‘almost perfect’ concordance (κw ≥ 0·81).

Yellow cells = ‘moderate’ concordance (κw = 0·41–0·60).

* NT and ACT both use the National School Canteen system.

#### Concordance across product categories

While pairwise concordance between guidelines ranged from moderate to almost perfect, concordance of product classifications varied across product categories (Table 2). ‘Vegetables’ (100 %) and ‘sweet snacks and desserts (78 %) had the highest concordance across all guidelines, indicating that all guidelines had adopted a relatively similar approach to classify these products. ‘Cold meals’ had the lowest concordance across all guidelines (0 %) due to a variety of approaches used in the guidelines and lack of clarity around the classification criteria. Additionally, ‘meat and seafood products’ (32 %), ‘dairy-based drinks’ (29 %) and ‘savoury snacks’ (23 %) had lower concordance compared to other product categories. For example, guidelines for healthcare facilities had slightly different criteria for savoury snacks with respect to energy and saturated fat content (Appendix II). Approximately half of the non-dairy drinks fell into the same healthiness categories across all guidelines, with the lowest concordance (11 %) observed in flavoured water products (without intense sweeteners) which fell into all healthiness categories (‘Green’, ‘Amber’ or ‘Red’) depending on the guideline used.

**Table 2.** Proportion of products falling into the same traffic light classification category by product type in an assessment of 17 Australian setting-specific nutrition guidelines in 2021–2023

Product category	Subcategories	*n* products	Across all settings	School	Health	Work
**Cold meals (e.g. salads, sandwiches)**		**40**	**0 %**	**0 %**	**0 %**	**63 %**
**Dairy-based drinks**		**90**	**29 %**	**73 %**	**38 %**	**79 %**
	Plain milk	39	59 %	100 %	59 %	100 %
	Flavoured milk	51	6 %	53 %	22 %	32 %
**Hot food/meals**		**154**	**62 %**	**77 %**	**87 %**	**95 %**
	Hot potato products	70	69 %	69 %	97 %	100 %
	Pies, rolls, pizza and savoury pastries	61	79 %	93 %	80 %	100 %
	Other heat-and-eat/convenience meals (excl. pizza, pies etc.)	23	0 %	61 %	74 %	74 %
**Meat and seafood products**		**107**	**33 %**	**37 %**	**49 %**	**80 %**
	Crumbed/coated seafood products	24	25 %	25 %	88 %	88 %
	Meat and chicken products (incl. meat alternatives)	23	43 %	48 %	48 %	52 %
	Processed meat (cold luncheon and cured meats)	20	70 %	85 %	70 %	70 %
	Seafood products (uncoated)	40	13 %	15 %	15 %	100 %
**Non-dairy drinks**		**108**	**52 %**	**73 %**	**74 %**	**84 %**
	Juice and fruit drinks	9	56 %	100 %	89 %	89 %
	Soft drinks	43	56 %	70 %	81 %	100 %
	Sports drinks	27	52 %	100 %	52 %	100 %
	Water (unflavoured)	11	100 %	100 %	100 %	100 %
	Water (flavoured)	18	11 %	11 %	67 %	11 %
**Savoury snacks**		**81**	**23 %**	**38 %**	**26 %**	**80 %**
	Chips and popcorn	42	21 %	21 %	26 %	91 %
	Nuts, legumes and nut mixes	9	44 %	78 %	44 %	56 %
	Savoury biscuits and crackers	30	20 %	50 %	20 %	73 %
**Sweet snacks/desserts**		**354**	**78 %**	**90 %**	**81 %**	**92 %**
	Cakes, slices and sweet tarts/pastries	77	94 %	95 %	96 %	97 %
	Confectionery	76	100 %	100 %	100 %	100 %
	Frozen desserts	97	69 %	80 %	76 %	91 %
	Yoghurt	18	33 %	94 %	33 %	100 %
	Other sweet snacks (non-confectionery, e.g. muesli bars)	86	65 %	86 %	66 %	81 %
**Vegetables**	**Frozen vegetables**	**33**	**100 %**	**100 %**	**100 %**	**100 %**

#### Alignment with national classification schemes

Of 653 products classified as ‘discretionary’ according to the ADG^(22)^, an average of 74 % were classified as ‘Red’ and 99 % either as ‘Red’ or ‘Amber’ across all traffic light-based systems. Of the products classified as ‘core’ according to the ADG (*n* 311), an average of 41 % were classified as ‘Green’ and 80 % either as ‘Green’ or ‘Amber’.

Of 754 products classified as ‘unhealthy/not recommended for promotion’ according to COAG guidelines^(30)^, an average of 69 % were classified as ‘Red’ and 97 % either as ‘Red’ or ‘Amber’ across all traffic light-based systems. Of the products that were allowed for promotion according to these guidelines (*n* 213), an average of 57 % were classified as ‘Green’ and 93 % either as ‘Green’ or ‘Amber’.

#### Concordance based on health star ratings

**Figure 4.**
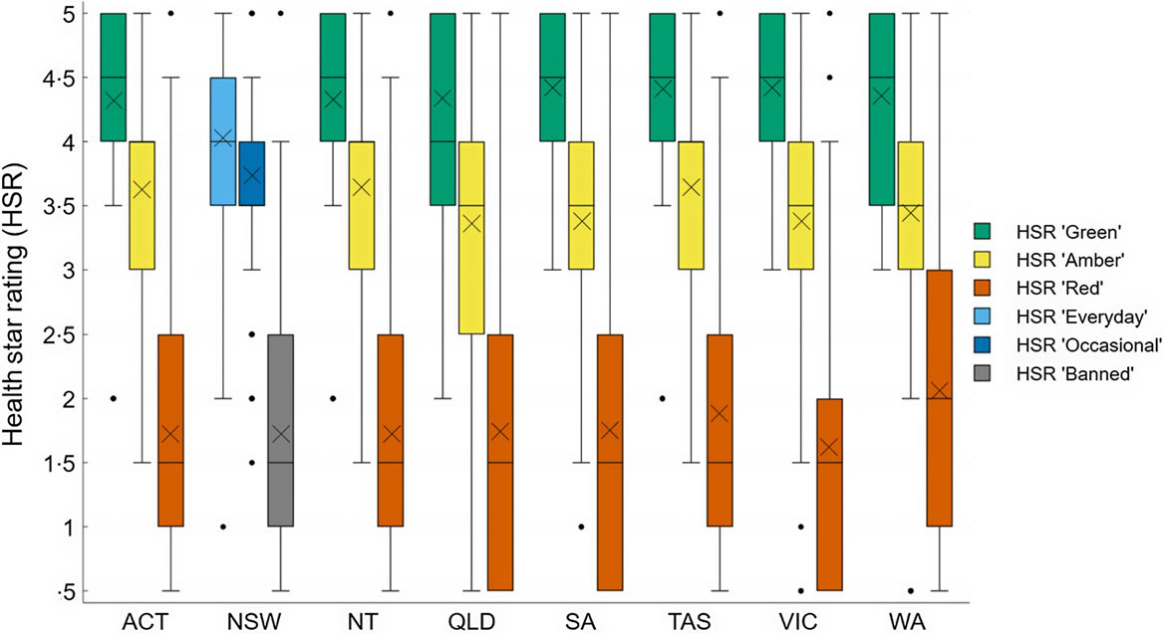
Health star rating comparison between guidelines for schools in an assessment of 17 Australian setting-specific nutrition guidelines in 2021–2023. Note that NT and ACT both use the National School Canteen classification system. X = mean HSR. HSR, health star rating.

**Figure 5.**
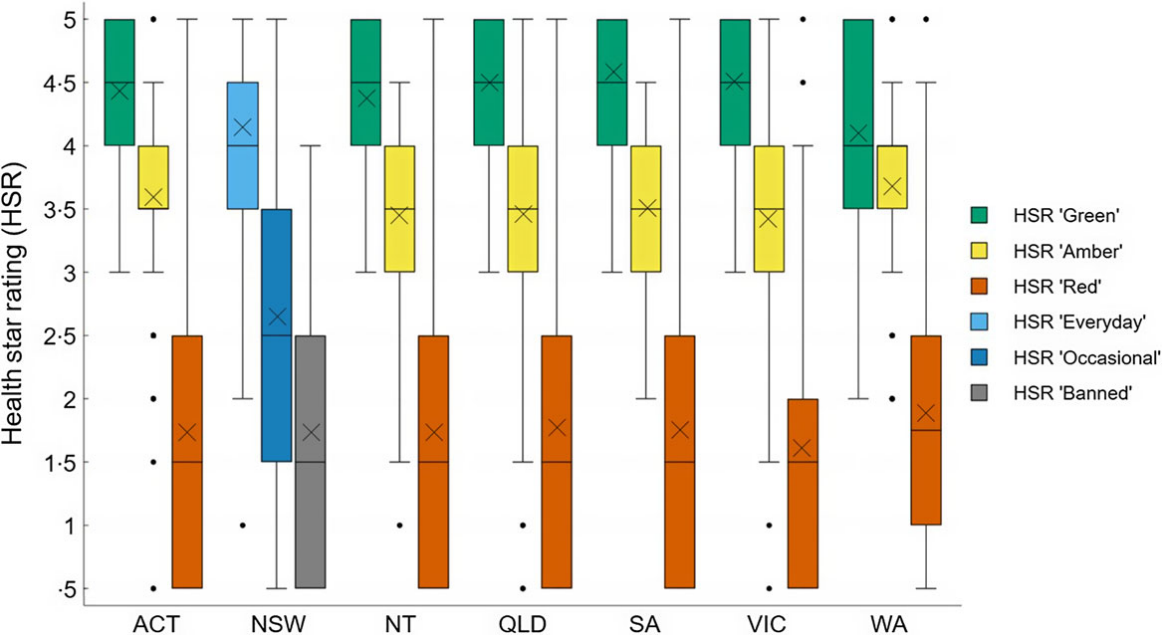
Health star rating comparison between guidelines for healthcare facilities in assessment of 17 Australian setting-specific nutrition guidelines in 2021–2023. X = mean HSR. HSR, health star rating.

Overall, products placed in similar healthiness categories across all guidelines using both traffic light-based and HSR classifications systems had similar HSR ratings (Figures 4 and 5). The HSR ranged between 2 to 5 for products classified as ‘Green’ or ‘Everyday’ and 0·5 to 5 for products in other healthiness categories. The mean HSR for ‘Green’ and ‘Everyday’ products was 4·3 (sd 0·67), ‘Amber’ and ‘Occasional’ products was 3·4 (sd 0·86) and ‘Red’ and ‘Banned’ products was 1·8 (sd 1·11) across all guidelines. However, the ANOVA results indicated significant differences in mean HSR across guidelines (e.g. the ‘Amber’ or ‘Occasional’ category for healthcare facilities, F(6, 2111) = 58·37, *P*< 0·001; and the ‘Red’ or ‘Banned’ category for schools, F(7, 4310) = 7·49, *P*< 0·001). Post-hoc comparisons using Tukey’s honestly significant difference test revealed that products placed in the ‘Occasional’ category according to the NSW guidelines for healthcare facilities had a lower mean HSR (x̄ = 2·6, sd = 1·24) compared to products classified as ‘Amber’ according to all other guidelines for this setting (Figure 5). The mean difference (MD) in the HSR for ‘Amber’ and ‘Occasional’ categories between NSW and other guidelines for healthcare settings ranged from MD = 0·82 (95 % CI (0·64, 1·00)) to MD = 1·07 (95 % CI (0·84, 1·30); see Appendix IV). This difference in mean HSR between NSW and other states was largely due to portion size being the only criterion to classify some ‘Occasional’ snacks such as confectionery, desserts and frozen desserts in NSW guidelines applying to healthcare facilities.

Additionally, the average HSR of products classified as ‘Red’ based on WA school guidelines was higher (x̄ = 2·1, sd 1·26) compared to products classified as ‘Red’ or ‘Banned’ according to other jurisdictions and guidelines (Figure 4). The mean difference in the HSR for ‘Red’ and ‘Banned’ categories between WA and other guidelines for schools ranged from MD = 0·23 (95 % CI (0·03, 0·43)) to MD = 0·45 (95 % CI (0·24, 0·65)). The complete results of all pairwise comparisons can be found in Appendix IV. The higher mean HSR of products classified as ‘Red’ based on WA school guidelines was mainly due to a higher number of products falling into the ‘Red’ category compared to other jurisdictions and guidelines. For example, WA school guidelines included a portion size criterion (≤ 100 g), while this criterion was higher in NSW guidelines (≤ 140 g) and absent from other guidelines.

## Discussion

This study examined the way that seventeen different food and drink classification guidelines for schools, workplaces and healthcare facilities across Australian jurisdictions classify commonly available products. Seven of eight Australian jurisdictions used a comparable food classification system (traffic light system) and 56 % of products were classified at the same level of healthiness across all guidelines using traffic light systems. The study found substantial pairwise concordance (Cohen’s κ mean 0·74) between those guidelines using a traffic light system, with most products falling into the same healthiness categories when comparing guidelines relating to the same setting (school, workplace or healthcare facility). The product classifications across different guidelines aligned with the HSR, with the average HSR of products classified as ‘Red’ being much lower compared to ‘Amber’ and ‘Green’. Almost all products classified as ‘discretionary’ according to the ADG (mean 99 %) and ‘unhealthy/not recommended for promotion’ according to the COAG guidelines (mean 97 %) were classified either as ‘Red’ or ‘Amber’ across all state-based guidelines, suggesting consensus among different schemes regarding unhealthy and discretionary food (i.e. foods not considered essential to a healthy diet^(22)^). However, on average, only 41 % of products classified as ‘core’ according to the ADG and 57 % of products allowed for promotion under COAG guidelines were classified as ‘Green’ according to state-based guidelines. This suggests potential disagreements between jurisdictions’ and national definitions of ‘healthy’ food (i.e. food considered essential for a healthy diet^(22)^).

Our findings align with previous literature^(18)^, confirming that Australian jurisdictions were still using different approaches to food classification guidelines within setting-specific nutrition policies from 2021–2023. Despite this, our findings indicate that product classification outcomes from two different state-based classification systems for the same setting type are likely to be similar, suggesting a foundational consistency in the classification criteria across Australian jurisdictions. This consistency suggests that any disagreements in classification criteria across jurisdictions may not be a significant barrier to harmonising the guidelines. Our findings are similar to those of Olstad et al.^(9)^ who conducted a similar study in public recreational and sporting settings in Canada and observed ‘moderate’ to ‘good’ concordance in food classification outcomes for three food classification systems used to assess the healthiness of products. However, our study highlights that there is likely to be limited agreement in classification outcomes when comparing all state-based guidelines simultaneously, suggesting a need for more consistent guidelines across states to reduce confusion and to support clearer messaging around healthy food choices across Australia.

Discrepancies in product classification outcomes between state-based guidelines mainly arose from variations in classification criteria and the grouping of products under different classification criteria. For example, ‘Uncoated seafood products’ (e.g. canned fish) were more likely to fall into less healthy classification categories in guidelines that did not have specified criteria for these products and these products were classified using the nutrition criteria for other ‘Meat and seafood products’. The lack of clarity around the classification outcomes (i.e. what products are considered ‘healthy’) might confuse manufacturers, distributors and retailers who attempt to make healthy changes in their product offering^(10,11)^, compromising goals to improve the healthiness of consumer purchases and dietary behaviour in government-owned settings. Our findings suggest that the lack of clarity arises especially from discrepancies between guidelines regarding whether a particular product is considered moderately healthy (‘Amber’) or least healthy (‘Red’).

Our findings confirm the concerns raised by Rosewarne et al.^(18)^ that there is variability in the food product groupings subject to different nutrient criteria between Australian jurisdictions. Rosewarne et al.^(18)^ suggested that more specific nutrient criteria targets may be needed for certain food categories to avoid healthier foods falling into less healthy classification categories, a suggestion that is also supported by the findings of the current study. In our study, differences in how products were grouped together in the classification guidelines resulted in certain products being subjected to stricter criteria, leading them to falling into less healthy categories in some jurisdictions compared to others. Vague product grouping may exclude options that can be considered nutritionally adequate but fall into ‘less healthy’ categories due to a lack of specific classification criteria appropriate for these products. Since stringent criteria may result in relatively few products being classified as ‘healthier’ in vaguely grouped categories (e.g. ‘Savoury snacks’, ‘Meat and seafood products’), external implementation support (e.g. from health promotion practitioners) is likely needed to help retailers identify products that meet the criteria and comply with the setting-based guidelines.^(10)^

A tension exists^(8)^ between detailed nutrition criteria for a multitude of product categories *v*. overly complex systems which is known to be a barrier to implementation and monitoring of healthy changes in food retail outlets^(10,11)^. Food-based criteria may be considered less complex and easier to comprehend and adhere to in certain contexts (e.g. food retail) compared to detailed nutrient-based classification systems^(6)^. However, future research could focus on determining how different classification systems (e.g. nutrient-based *v*. food-based) perform in different public settings. This could involve testing different systems to understand which one is the most feasible to implement, helping to identify the most effective and practical systems for promoting healthy food choices in these settings.

Given the moderate to substantial pairwise concordance yet limited overall agreement in classification outcomes across all state-based guidelines, it may be beneficial to introduce a nationally cohesive classification system. A national system could be adapted to settings catering to target population groups with specific nutritional needs (e.g. schools). While some evidence suggests that different food classification schemes may be needed for different purposes (e.g. school nutrition policies *v*. regulation of food marketing)^(12,13)^, it would be beneficial to aim for harmonisation across schemes that serve the same purpose. All state-based guidelines in Australia are underpinned by the ADG; however, state and territory governments hold the responsibility of regulating their own public settings, resulting in different state-specific guidelines^(18)^. Additionally, varying policy cycles across jurisdictions result in discrepancies in the recency of updates among the guidelines. More cohesive guidelines would provide retailers, manufacturers and suppliers with clearer direction on which products are permitted for sale in government-owned settings, supporting retailers to implement healthy changes in their product offerings; a need that has been highlighted in the previous research^(18,23)^. National guidelines could help retailers, distributors and manufacturers implement healthier changes as they often struggle with the complexity and multitude of institutional level food and drink classification guidelines^(10,11)^. Adopting national guidelines could additionally prevent duplication of efforts and optimise resource allocation when developing implementation support and training packages^(11)^. However, there is a risk that efforts to harmonise might result in adoption of the weakest standards. For this reason, it is imperative that decisions on appropriate classification criteria are evidence-based and aligned with desired public health outcomes, ensuring long-term benefits for population health. As a first step, this study has reviewed and synthesised criteria and categories used in the current food classification guidelines. The next step would involve bringing jurisdictions together to negotiate and agree on the most appropriate criteria to adopt for a national approach. The current revision of the ADG^(40)^ provides an opportunity for jurisdictions to adopt a more cohesive national approach.

National or more cohesive guidelines would also facilitate greater consistency in the monitoring of the healthiness of food outlet environments across Australia. There is limited evidence on the successful implementation of government nutrition policies in food outlets within government-owned settings. Most research has focused on schools where compliance is mandatory^(41)^ and adherence to these policies is rarely systematically monitored^(42)^. Monitoring tools that enable systematic monitoring across states could potentially improve adherence. Many existing food environment monitoring tools use food-based classification systems based on national dietary guidelines or use key indicators (e.g. presence of deep-fried foods or sugar-sweetened drinks)^(2)^, those that wish to report on state-based guidelines usually report against the specific guidelines for their jurisdiction and setting (e.g. healthcare). For example, the ‘Healthy choices’ policy directive for Victorian public health services mandates that all food retail outlets in publicly funded healthcare settings must adhere to the product frequency targets specified in the Victorian Healthy Choices Guidelines (Appendix I)^(36,43)^. The findings of the current study can be used to guide the development of food outlet monitoring tools to be used across different settings and jurisdictions, including by those with limited nutrition knowledge (e.g. food outlet staff). Findings also suggest that a national or more cohesive approach would provide an opportunity for more efficient development and scalability of tools and resources for Australian nutritionists, health promotion practitioners and retailers.

### Strengths and limitations

To our knowledge, this is the first study to investigate the impact of variations in jurisdictional food classification guidelines for different government-owned settings (schools, healthcare facilities and workplaces) on food product classifications and the first to investigate the level of concordance between such guidelines in Australia. One of its key strengths is the assessment of guidelines according to how they classify a large sample of products available for sale in Australia, across multiple product categories. The database of state food classification guidelines which was updated for this study can also be a useful resource for researchers, manufacturers, and policymakers. One of the limitations of this study is the relatively high proportion of sweet snacks (37 %) included in the product sample, driven by the inclusion of real-world products. While the product sample is likely representative of the prevalent products in Australian government-owned food outlets, surveying actual product availability in a sample of public schools, healthcare settings and workplaces could provide an alternative approach. Another limitation is that the real-world sample used in the current study was drawn from government-owned sporting settings, potentially introducing bias due to differences in food offerings compared to the settings of the guidelines included in the study. Finally, the limitation of the Cohen’s Kappa statistic to interpret the results is that the cut-off points are not based on objective criteria^(31)^.

### Conclusion

While the classification of product healthiness is largely similar across the traffic light system-based guidelines in Australian jurisdictions, this study highlighted that discrepancies for some product categories exist across the guidelines. The complexity and number of different guidelines may cause confusion to users of these guidelines (e.g. retailers, manufacturers) and hinder the implementation and monitoring of healthy changes in food outlet product offerings. Evidence-based national food and drink classification guidelines for food outlets in government-owned settings would offer several advantages. It would provide clarity, minimise duplication of implementation efforts and resources required from the state and territory governments and ensure consistency across jurisdictions in assessing and monitoring the healthiness of these outlets across Australia. Cohesive, setting-specific nutrition policies across Australian jurisdictions would ultimately help ensure clarity for food businesses on how to better support population health.

## Supporting information

Backman et al. supplementary material 1 Backman et al. supplementary material

Backman et al. supplementary material 2 Backman et al. supplementary material

Backman et al. supplementary material 3 Backman et al. supplementary material

Backman et al. supplementary material 4 Backman et al. supplementary material

## Supplementary material

For supplementary material accompanying this paper visit https://doi.org/10.1017/S1368980025000059
