# Supplementary material for: Concordance of Australian state and territory government guidelines for classifying the healthiness of foods in public settings
Source: Public Health Nutr. 2025 Feb 3;28(1):e47. doi: 10.1017/S1368980025000059 (PMC11983995; doi:10.1017/S1368980025000059)
Supplement: Backman et al. supplementary material 1 — Backman et al. supplementary material [file S1368980025000059sup001.docx]

**Appendix III: Product types and categories included in assessment of 17 Australian setting-specific nutrition guidelines for schools, workplaces, and health care facilities in 2021-2023**

| **Product category** | **Sub-categories** | **n Products** | **Proportion of products (%)** |
| --- | --- | --- | --- |
| Cold meals ^[[1]](#footnote-2)^ | Cold ready-to-eat meals (e.g., salads, sandwiches) ^a^ | **40** | **4%** |
| Dairy-based drinks | Plain milk ^b^ | 39 | 4% |
|  | Flavoured milk | 51 | 5% |
|  | Dairy-based drinks (all) | **90** | **9%** |
| Hot food / meals | Hot potato products | 70 | 7% |
|  | Pies, rolls, pizza and savoury pastries | 61 | 6% |
|  | Other heat-and-eat/convenience meals (excl. pies, rolls etc.) ^b^ | 23 | 2% |
|  | Hot food / meals (all) | **154** | **16%** |
| Meat and seafood products ^[[2]](#footnote-3)^ | Crumbed/coated seafood products | 24 | 2% |
|  | Meat products (incl. meat alternatives) | 23 | 2% |
|  | Processed meat (cold luncheon and cured meats) | 20 | 2% |
|  | Uncoated seafood products | 40 | 4% |
|  | Meat and seafood products (all) | **107** | **11%** |
| Non-dairy drinks | Juice and fruit drinks | 9 | 1% |
|  | Soft drinks | 43 | 4% |
|  | Sports drinks | 27 | 3% |
|  | Water (unflavoured) | 11 | 1% |
|  | Water (flavoured) | 18 | 2% |
|  | Non-dairy drinks (all) | **108** | **11%** |
| Savoury snacks | Chips and popcorn | 42 | 4% |
|  | Nuts, legumes and nut mixes | 9 | 1% |
|  | Savoury biscuits and crackers | 30 | 3% |
|  | Savoury snacks (all) | **81** | **8%** |
| Sweet snacks / desserts | Cakes, slices and sweet tarts/pastries | 77 | 8% |
|  | Confectionery | 76 | 8% |
|  | Frozen desserts | 97 | 10% |
|  | Yoghurt | 18 | 2% |
|  | Other sweet snacks (non-confectionery e.g., muesli bars, biscuits) | 86 | 9% |
|  | Sweet snacks / desserts (all) | **354** | **37%** |
| Vegetables ^b^ | Frozen vegetables | **33** | **3%** |
| Top-selling brands | | **794** | **82%** |
| Real-world | | **173** | **18%** |
| **All products** | | **967** |  |

1. Category only included in the sample of products **obtained from real-world food outlets**. [↑](#footnote-ref-2)
2. Category only included in the sample of products representing **top-selling food brands**. [↑](#footnote-ref-3)
