# Supplementary material for: Concordance of Australian state and territory government guidelines for classifying the healthiness of foods in public settings
Source: Public Health Nutr. 2025 Feb 3;28(1):e47. doi: 10.1017/S1368980025000059 (PMC11983995; doi:10.1017/S1368980025000059)
Supplement: Backman et al. supplementary material 2 — Backman et al. supplementary material [file S1368980025000059sup002.docx]

**Appendix II: Summary of the strictest^[[1]](#footnote-2)^ ‘Amber’ product nutrient criteria within key food product categories across settings and jurisdictions^^[[2]](#footnote-3)^^**

| **Food category** | | **Energy** | **Saturated Fat** | **Sodium** | **Sugar** | **Fibre** | **Other** |
| --- | --- | --- | --- | --- | --- | --- | --- |
| **Cold meals** | **Cold ready-to-eat meals (e.g., salads, sandwiches)** | Variations in the classification approach (classified mainly based on major ingredients); strictest criteria difficult to determine. | | | | | |
| **Dairy-based drinks** | **Plain milk** | - | - | - | - | - | Full fat |
|  | **Flavoured milk** | All:  ≤1600 kJ /serve | - | - | - | - | All:  No added confectionery or ice cream  Health:  Portion size ≤500mL  Reduced fat  Schools:  Portion size ≤375mL  No coffee flavoured varieties |
| **Hot food / meals** | **Heat-and-eat meals (excl. pies, rolls etc.)** | Health:  ≤2500kJ/  serve    Schools:  ≤1000kJ/  100g  and  ≤2500kJ/  serve | All:  ≤2g/100g | All:  ≤300mg/100g  and  ≤900mg/serve | - | All:  ≥3g/  serve | Health:  -    Schools:  Portion size ≤300g |
|  | **Hot potato products** | All:  ≤1000kJ/ 100g | All:  ≤5g/ 100g | All:  ≤400mg/100g | - | - | All:  No deep-fried varieties    Health:  Portion size ≤150g    Schools:  Portion size ≤100g |
|  | **Pies, rolls, pizza and savoury pastries** | All:  ≤1000kJ/ 100g | All:  ≤5g/ 100g | All:  ≤400mg/ 100g | - | - | All:  No deep-fried varieties    Health:  Portion size ≤250g    Schools:  Portion size ≤200g |
| **Meat and seafood products** | **Crumbed seafood products** | All:  ≤1000kJ/ 100g | All:  ≤5g/ 100g | All:  ≤450mg/ 100g | - | - | All:  No deep-fried varieties    Health:  Portion size ≤150g    Schools:  Portion size ≤100g |
|  | **Meat products (incl. meat alternatives)** | All:  ≤1000kJ/ 100g | All:  ≤5g/ 100g | All:  ≤450mg/ 100g | - | - | All:  No deep-fried varieties    Health:  Portion size ≤150g    Schools:  Portion size ≤100g |
|  | **Processed meat (cold luncheon and cured meats)** | Health:  ≤900kJ/ 100g    Schools:  ≤1000kJ/ 100g | All:  ≤3g/ 100g | Health:  ≤750mg/ 100g    Schools:  ≤450mg/ 100g | - | - | All:  Portion size ≤50g    Health (WA):  Processed poultry meats only (no ‘Red’ meats) |
|  | **Seafood products (uncoated)** | Health:  Fish canned in oil, 1000kJ/ 100g | Health:  Fish canned in oil, ≤5g/ 100g | Health:  Fish canned in oil, ≤450mg/ 100g | - | - | All:  Smoked salmon/trout classified as 'Red' in some jurisdictions    Health:  Fish canned in oil, portion size ≤150g    Schools:  Canned fish in brine, oil or flavoured sauce |
| **Non-dairy drinks** | **Juice and fruit drinks** | - | - | - | - | - | All:  ≥99% fruit or vegetable juice    Portion size ≤250mL |
|  | **Soft drinks** | - | - | - | - | - | All:  Sugar-free varieties    Portion size ≤600mL |
|  | **Sports drinks** | - | - | - | - | - | Classified as 'Red' in some jurisdictions |
|  | **Water (flavoured)** | - | - | - | - | - | All:  No added sugar or intense sweeteners  Schools (QLD and WA only): Classified as ‘Red’ |
|  | **Water (unflavoured)** | - | - | - | - | - | Classified as ‘Green’ in all jurisdictions and settings |
| **Savoury snacks** | | All:  ≤600kJ/ serve  and 1800kJ/ 100g | Health:  ≤2g/ serve (ACT, SA, VIC)    ≤3g/ serve (QLD)    ≤3g/ 100g (NT; WA for popcorn and legume snacks)    ≤5g/100g (WA for chips, crackers, savoury biscuits, crispbreads and fruit chips)    Schools:  ≤2g/ serve | Health:  ≤120mg/ 100g (WA for popcorn and legume snacks)    ≤200mg/ serve (ACT, NT, QLD, SA, VIC)    ≤400mg/ 100g (WA for chips, crackers, savoury biscuits, crispbreads and fruit chips)    Schools:  ≤200mg/ serve | Health:  Popcorn and legume snacks only,  <20g/ 100g (WA) | - | All:  No added confectionery |
| **Sweet snacks / desserts** | **Cakes, slices and sweet tarts/pastries** | All:  ≤900kJ/ serve | All:  ≤3g/ serve | - | - | All:  ≥1.5g/ serve | Health (WA only):  Only varieties containing fruit and/or vegetables can be classified as ‘Amber’  Portion size ≤60g |
|  | **Confectionery** | - | - | - | - | - | Classified as 'Red' in all jurisdictions |
|  | **Frozen desserts** | All:  ≤600kJ/ serve | All:  ≤2g/ 100g    And    ≤3g/ serve | - | Health (WA only):  ≤15g/ 100g | - | All:  'Red' if contains confectionery (e.g., chocolate, caramel, biscuit, crumble)  Health (WA only):  Dairy-free ice cream, gelato, sorbet and ice blocks are classified as 'Red' |
|  | **Other sweet snacks (e.g. muesli bars, biscuits)** | All:  ≤600kJ/ serve | All:  ≤3g/ serve | - | Health (WA only):  ≤20g/100g | All:  ≥1g/ serve | All:  No added confectionery |
|  | **Yoghurt** | - | - | - | - | - | All:  Full fat varieties (reduced-fat varieties are Green)  No added confectionery |

1. The term 'strictest criteria' refers to the specific criteria a product must meet to be classified as 'Amber' (or healthier) across all guidelines for schools and health facilities included in this study. Using the strictest criteria for 'Amber' products represents the middle ground between the healthiest ('Green') and least healthy ('Red') options, highlighting the most conservative classification criteria that products must meet to avoid falling into the least healthy ‘Red’ category according to all traffic light-based guidelines. [↑](#footnote-ref-2)
2. Setting and jurisdictions included *Schools* - ACT, NT, QLD, SA, TAS, VIC, WA; *Health facilities* - ACT, NT, QLD, SA, VIC, WA

   ACT=Australian Capital Territory, NT=Northern Territory, QLD=Queensland, SA=South Australia, TAS=Tasmania, VIC=Victoria, WA=Western Australia [↑](#footnote-ref-3)
