# Supplementary material for: Concordance of Australian state and territory government guidelines for classifying the healthiness of foods in public settings
Source: Public Health Nutr. 2025 Feb 3;28(1):e47. doi: 10.1017/S1368980025000059 (PMC11983995; doi:10.1017/S1368980025000059)
Supplement: Backman et al. supplementary material 3 — Backman et al. supplementary material [file S1368980025000059sup003.docx]

**Appendix IV: Pairwise comparisons of mean Health Star Ratings (HSR) for each healthiness category between guidelines (Tukey HSD Test)**

|  |  | | ‘Green’ or ‘Everyday’ | | | | ‘Amber’ or ‘Occasional’ | | | | ‘Red’ or ‘Banned’ | | |
| --- | --- | --- | --- | --- | --- | --- | --- | --- | --- | --- | --- | --- | --- |
| Setting | Guidelines compared^[[1]](#footnote-1)^ | | Mean Difference | | 95% Confidence Interval | | Mean Difference | | 95% Confidence Interval | | Mean Difference | 95% Confidence Interval | |
| *Schools* | NT vs ACT | | 0.00 | | [-0.23, 0.23] | | 0.00 | | [-0.22, 0.22] | | 0.00 | [-0.21, 0.21] | |
|  | QLD vs ACT | | -0.12 | | [-0.36, 0.11] | | -0.25 | | [-0.46, -0.05] ***** | | 0.02 | [-0.19, 0.23] | |
|  | SA vs ACT | | 0.09 | | [-0.15, 0.34] | | -0.22 | | [-0.43, -0.02] ***** | | -0.01 | [-0.22, 0.20] | |
|  | VIC vs ACT | | 0.13 | | [-0.11, 0.37] | | -0.22 | | [-0.42, -0.02] ***** | | -0.14 | [-0.35, 0.07] | |
|  | WA vs ACT | | 0.02 | | [-0.23, 0.27] | | -0.17 | | [-0.39, 0.06] | | 0.31 | [ 0.11, 0.51] ***** | |
|  | TAS vs ACT | | 0.11 | | [-0.14, 0.36] | | 0.02 | | [-0.20, 0.23] | | 0.08 | [-0.12, 0.28] | |
|  | ACT vs NSW | | 0.27 | | [0.06, 0.48] ***** | | 0.12 | | [-0.36, 0.12] | | 0.00 | [-0.21, 0.20] | |
|  | NT vs NSW | | 0.27 | | [ 0.06, 0.49] ***** | | -0.12 | | [-0.36, 0.12] | | 0.00 | [-0.21, 0.20] | |
|  | QLD vs NSW | | 0.15 | | [-0.06, 0.36] | | -0.38 | | [-0.60, -0.15] ***** | | 0.02 | [-0.19, 0.22] | |
|  | SA vs NSW | | 0.37 | | [ 0.14, 0.59] ***** | | -0.35 | | [-0.57, -0.12] ***** | | -0.01 | [-0.22, 0.20] | |
|  | VIC vs NSW | | 0.40 | | [ 0.18, 0.62] ***** | | -0.34 | | [-0.56, -0.12] ***** | | -0.14 | [-0.35, 0.07] | |
|  | WA vs NSW | | 0.29 | | [ 0.06, 0.52] ***** | | -0.29 | | [-0.53, -0.05] ***** | | 0.31 | [ 0.11, 0.51] ***** | |
|  | TAS vs NSW | | 0.38 | | [ 0.15, 0.61] ***** | | -0.10 | | [-0.34, 0.13] | | 0.08 | [-0.12, 0.28] | |
|  | QLD vs NT | | -0.12 | | [-0.36, 0.11] | | -0.25 | | [-0.46, -0.05] ***** | | 0.02 | [-0.19, 0.23] | |
|  | SA vs NT | | 0.09 | | [-0.15, 0.34] | | -0.22 | | [-0.43, -0.02] ***** | | -0.01 | [-0.22, 0.20] | |
|  | VIC vs NT | | 0.13 | | [-0.11, 0.36] | | -0.22 | | [-0.42, -0.02] ***** | | -0.14 | [-0.35, 0.07] | |
|  | WA vs NT | | 0.02 | | [-0.23, 0.27] | | -0.17 | | [-0.39, 0.06] | | 0.31 | [ 0.11, 0.51] ***** | |
|  | TAS vs NT | | 0.11 | | [-0.14, 0.36] | | 0.02 | | [-0.20, 0.23] | | 0.08 | [-0.12, 0.28] | |
|  | SA vs QLD | | 0.22 | | [-0.02, 0.46] | | 0.03 | | [-0.16, 0.22] | | -0.02 | [-0.24, 0.19] | |
|  | VIC vs QLD | | 0.25 | | [ 0.01, 0.49] ***** | | 0.04 | | [-0.15, 0.22] | | -0.15 | [-0.37, 0.06] | |
|  | WA vs QLD | | 0.14 | | [-0.10, 0.39] | | 0.09 | | [-0.13, 0.30] | | 0.29 | [ 0.09, 0.50] ***** | |
|  | TAS vs QLD | | 0.23 | | [-0.02, 0.48] | | 0.27 | | [ 0.07, 0.48] ***** | | 0.06 | [-0.14, 0.27] | |
|  | VIC vs SA | | 0.03 | | [-0.22, 0.28] | | 0.04 | | [-0.17, 0.18] | | -0.15 | [-0.35, 0.07] | |
|  | WA vs SA | | -0.07 | | [-0.33, 0.19] | | 0.06 | | [-0.15, 0.26] | | 0.29 | [ 0.09, 0.50] ***** | |
|  | TAS vs SA | | 0.01 | | [-0.25, 0.27] | | 0.24 | | [ 0.04, 0.44] ***** | | 0.09 | [-0.12, 0.30] | |
|  | WA vs VIC | | -0.11 | | [-0.36, 0.15] | | 0.05 | | [-0.15, 0.26] | | 0.45 | [ 0.24, 0.65] ***** | |
|  | TAS vs VIC | | -0.02 | | [-0.27, 0.24] | | 0.24 | | [ 0.04, 0.43] ***** | | 0.22 | [ 0.01, 0.43] ***** | |
|  | WA vs TAS | | -0.09 | | [-0.35, 0.18] | | -0.19 | | [-0.41, 0.03] | | 0.23 | [0.03, 0.43] ***** | |
|  |  |  | |  | |  | |  | |  | | |  |
| *Healthcare facilities* | NT vs ACT | | -0.10 | | [-0.33, 0.13] | | -0.13 | | [-0.33, 0.08] | | -0.00 | [-0.20, 0.20] | |
|  | QLD vs ACT | | 0.06 | | [-0.18, 0.31] | | -0.11 | | [-0.32, 0.09] | | 0.05 | [-0.14, 0.25] | |
|  | SA vs ACT | | 0.12 | | [-0.12, 0.36] | | -0.09 | | [-0.30, 0.12] | | 0.00 | [-0.19, 0.20] | |
|  | VIC vs ACT | | 0.04 | | [-0.20, 0.28] | | -0.13 | | [-0.33, 0.07] | | -0.08 | [-0.28, 0.12] | |
|  | WA vs ACT | | -0.35 | | [-0.57, -0.14] * | | 0.12 | | [-0.12, 0.37] | | 0.15 | [-0.04, 0.35] | |
|  | ACT vs NSW | | 0.34 | | [0.14, 0.55] * | | 0.95 | | [0.75, 1.15] * | | 0.05 | [-0.17, 0.27] | |
|  | NT vs NSW | | 0.24 | | [0.05, 0.44] * | | 0.83 | | [0.63, 1.02] * | | 0.05 | [-0.18, 0.27] | |
|  | QLD vs NSW | | 0.41 | | [0.19, 0.62] * | | 0.84 | | [0.65, 1.03] * | | 0.10 | [-0.12, 0.32] | |
|  | SA vs NSW | | 0.47 | | [0.26, 0.68] * | | 0.86 | | [0.67, 1.06] * | | 0.05 | [-0.17, 0.27] | |
|  | VIC vs NSW | | 0.39 | | [0.18, 0.59] * | | 0.82 | | [0.64, 1.00] * | | -0.03 | [-0.25, 0.20] | |
|  | WA vs NSW | | -0.01 | | [-0.19, 0.17] | | 1.07 | | [0.84, 1.30] * | | 0.20 | [-0.01, 0.42] | |
|  | QLD vs NT | | 0.16 | | [-0.07, 0.40] | | 0.01 | | [-0.19, 0.21] | | 0.06 | [-0.14, 0.25] | |
|  | SA vs NT | | 0.22 | | [-0.01, 0.46] | | 0.04 | | [-0.17, 0.24] | | 0.00 | [-0.19, 0.20] | |
|  | VIC vs NT | | 0.14 | | [-0.09, 0.38] | | -0.01 | | [-0.20, 0.19] | | -0.08 | [-0.28, 0.13] | |
|  | WA vs NT | | -0.25 | | [-0.45, -0.05] * | | 0.25 | | [0.01, 0.49] * | | 0.16 | [-0.04, 0.35] | |
|  | SA vs QLD | | 0.06 | | [-0.19, 0.31] | | 0.02 | | [-0.18, 0.22] | | -0.05 | [-0.25, 0.15] | |
|  | VIC vs QLD | | -0.02 | | [-0.27, 0.22] | | -0.01 | | [-0.21, 0.17] | | -0.13 | [-0.33, 0.07] | |
|  | WA vs QLD | | -0.42 | | [-0.64, -0.20] * | | 0.23 | | [0.00, 0.49] | | 0.10 | [-0.09, 0.29] | |
|  | VIC vs SA | | -0.08 | | [-0.32, 0.16] | | -0.04 | | [-0.24, 0.15] | | -0.13 | [-0.33, 0.07] | |
|  | WA vs SA | | -0.47 | | [-0.69, -0.26] * | | 0.21 | | [-0.03, 0.45] | | 0.15 | [-0.04, 0.34] | |
|  | WA vs VIC | | -0.39 | | [-0.61, -0.18] * | | 0.25 | | [0.02, 0.49] * | | 0.23 | [0.04, 0.43] * | |

1. ACT=Australian Capital Territory, NSW,=New South Wales, NT=Northern Territory, QLD=Queensland, SA=South Australia, TAS=Tasmania, VIC=Victoria, WA=Western Australia

   * Difference in mean HSR is statistically significant [↑](#footnote-ref-1)
