# Supplementary material for: Concordance of Australian state and territory government guidelines for classifying the healthiness of foods in public settings
Source: Public Health Nutr. 2025 Feb 3;28(1):e47. doi: 10.1017/S1368980025000059 (PMC11983995; doi:10.1017/S1368980025000059)
Supplement: Backman et al. supplementary material 4 — Backman et al. supplementary material [file S1368980025000059sup004.docx]

**Appendix I: List of state and territory procurement policies for inclusion and comparison of the criteria relating to product availability placement, pricing and promotion of ‘Green’, ‘Amber’ and ‘Red’ or ‘Everyday’ and ‘Occasional’ products**

| Jurisdiction and year introduced or updated | Name of Procurement Policy/ Nutrient Standards | System | Criteria for | | | |
| --- | --- | --- | --- | --- | --- | --- |
|  |  |  | Frequency of different foods and drinks permitted for food outlets | Product placement | Pricing | Promotion |
| Public schools |  |  |  |  |  |  |
| Australian Capital Territory, 2017 | ACT Public School Food and Drink Policy ^(^[^1^](#_ENREF_1)^)^ / The National Healthy School Canteen Guidelines ^(^[^2^](#_ENREF_2)^)^ | Traffic light | ‘Green’: Encouraged and promoted  ‘Amber’: Should not dominate the choices. Should be avoided in large serving sizes.  ‘Red’: Should not be available. Sugary drinks banned. | ‘Green’ products should be well-presented. | ‘Green’ products should be the focus of meal deals. | ‘Green’ products should be promoted and well-presented. |
| New South Wales, 2020 | The NSW Healthy School Canteen^(^[^3^](#_ENREF_3)^)^ Strategy Food And Drink Criteria ^(^[^3^](#_ENREF_3)^)^ | HSR | ‘Everyday’: ≥ 75%  ‘Occasional’: < 25%  ‘Banned’: Should not be available | ‘Everyday’ foods and drinks  in prominent positions. | ‘Everyday’ items should be great value for-money.    Only include ‘Everyday’ foods and drinks in meal deals or specials offers. | Promote only ‘Everyday’ foods and drinks on menu boards, signage, posters, school newsletters and websites. |
| Northern Territory, 2017 | School Nutrition and Healthy  Eating Policy ^(^[^4^](#_ENREF_4)^)^/ The National Healthy School Canteen Guidelines ^(^[^2^](#_ENREF_2)^)^ | Traffic light | ‘Green’: Main choice on canteen menus. Large variety of these foods and drinks must be available every day.  ‘Amber’: Must not dominate the menu.  ‘Red’: Not to be sold or provided in schools (unless part of a whole school event) | ‘Green’ products should be well-presented. | ‘Green’ products should be the focus of meal deals. | ‘Green’ products should be promoted and well-presented. |
| Queensland, 2020 | Smart Choices Healthy Food and Drink Supply for Queensland Schools, v1.4 ^(^[^5^](#_ENREF_5)^)^ | Traffic light | ‘Green’: Encouraged and promoted  ‘Amber’: Should not dominate the choices. Should be avoided in large serving sizes.  ‘Red’: Not to be sold in school canteens. Some may be provided on a maximum of two occasions a term in certain situations. | N/A | N/A | ‘Green’ products should be promoted and encouraged. |
| South Australia, 2023 | Food and Drink Classification Guide for South Australian Public Schools^(^[^6^](#_ENREF_6)^)^ | Traffic light | ‘Green’: Should make up the majority of choices (≥ 60% recommended).  ‘Amber’: Should not dominate the choices. Should be avoided in large serving sizes.  ‘Red 1’: Limit availability to twice per term. Otherwise, should not be sold in school canteens.  ‘Red 2’: Should not be sold in school canteens. | ‘Green’ products should be well-presented.    ‘Green’ products should be placed at eye level on shelves and at the front of cabinets and bain-maries.    ‘Green’ options should be placed at the top of the menu.    ‘Green’ products should be placed in high traffic areas.    ‘Amber’ products should not be displayed at prominent positions at the expense of ‘Green’ options. | ‘Green’ foods and drinks are favourably priced (where possible).    ‘Green’ products should be the focus of special offers such  as meal deals, combos, and loyalty cards. | ‘Amber’ foods and drinks should not be promoted at the expense of ‘Green’ options.    ‘Green’ products should be promoted and encouraged.    ‘Red’ 1 food and drinks should not be promoted or encouraged. |
| Tasmania, 2021 | School Food Matters, Smart Food Guide, a Whole School Approach to Eating Well^(^[^7^](#_ENREF_7)^)^ | Traffic light | Tasmania ranks schools based on the proportions of ‘Green’/’Amber’/’Red’ products on their school food-service menu (Platinum, Gold, Silver, Bronze).  Platinum: ≥ 80% ‘Green’ items and no ‘Red’ items; ≥2 fruit or vegetable snacks available. Sustainability focus.  Gold: ≥ 60% ‘Green’ items and no ‘Red’ items.  Silver: ≥ 50% ‘Green’ items and < 10% ‘Red’ items.  Bronze: ≥ 40% ‘Green’ items and < 20% ‘Red’ items (< 8 varieties, < 3 confectionery varieties). | ‘Green’ options should be placed at eye level.    ‘Green’ options should be available in multiple places to encourage optimal sales. | ‘Green’ options should be affordable and preferably cheaper than ‘Amber’ or ‘Red’ options. | Promote healthier options actively. |
| Victoria, 2012 | Healthy Canteen Kit^(^[^8^](#_ENREF_8)^)^ | Traffic light | ‘Green’: ≥ 50%, ‘Amber’: < 50%  ‘Red’: Should not be available | ‘Green’ options should be made easy for students to choose.    ‘Amber’ options should not dominate the menu.    ‘Red’ options should not be regularly available or displayed in school areas. | ‘Amber’ items can be made ‘Greener’ by partnering with ‘Green’ options (meal deals). | ‘Green’ options should be included as the main choices.    ‘Amber’ options should be offered in smaller serving sizes. |
| Western Australia, 2023 | Student Health in Public Schools Policy^(^[^9^](#_ENREF_9)^)^ / The Star Choice™ Nutrient Criteria^(^[^10^](#_ENREF_10)^)^ (previously FOCIS Nutrient Criteria^(^[^11^](#_ENREF_11)^)^)* | Traffic light | ‘Green’: ≥ 60%, ‘Amber’: < 40%  ‘Red’: Should not be available | N/A | N/A | A wide range of healthy food should be promoted. |
| Workplaces |  |  |  |  |  |  |
| Northern Territory, 2017 | The Healthy Workplace  Toolkit ^(^[^12^](#_ENREF_12)^)^ | Traffic light | ‘Green’: ≥50%, ‘Red’: < 20% | Place healthier options at eye level in vending machines. | Water should always be available and free of charge (e.g., tap water, bubblers).    Healthier products should be comparable in price to less healthy options. | A wide variety of ‘Green’ options should be promoted.    Healthier food alternatives or non-food fundraisers should be encouraged. |
| Victoria, 2020 | Healthy choices: Healthy Eating Policy and Catering Guide For Workplaces, 2016 ^(^[^13^](#_ENREF_13)^)^ / Healthy Choices: Food and Drink Classification Guide, 2020 ^(^[^14^](#_ENREF_14)^)^ | Traffic light | ‘Green’: ≥ 50%, ‘Red’: < 20% | N/A | N/A | ‘Green’ options should be promoted and encouraged.    ‘Amber’ options should not be promoted at the expense of ‘Green’ choices.    ‘Red’ should not be included in meal or point of sale promotions. |
| Health facilities |  |  |  |  |  |  |
| Australian Capital Territory, 2016 | Healthy Food and Drink Choices Policy ^(^[^15^](#_ENREF_15)^)^ | Traffic light | ‘Green’ & ‘Amber’: ≥ 80%  ‘Red’: < 20% | ‘Red’ or ‘Amber’ foods and drinks should not be placed in prominent areas or at eye level. | N/A | Only ‘Green’ options can be advertised or promoted.    ‘Red’ or ‘Amber’ options should not be advertised or promoted.    The ACT Government logo should not be used alongside ‘Red’ or ‘Amber’ category options. |
| New South Wales, 2017 | Healthy Food and Drink in NSW Health Facilities for Staff and Visitors Framework ^(^[^16^](#_ENREF_16)^)^ | HSR | ‘Everyday’: ≥ 75%  ‘Occasional’: < 25%  ‘Banned’: Should not be available. Sugary drinks should not be sold. | ‘Everyday’ options should be placed in prominent locations | Value pricing should highlight ‘Everyday’ foods and drinks.    Include ‘Everyday’ foods and drinks in meal deals (e.g., coffee + snack deal) | Promotional activities should highlight ‘Everyday’ products. |
| Northern Territory, 2017 | Healthy Choices Made Easy -  Healthy Food and Drink Options for Staff, Volunteers and Visitors in NT Heath Facilities Policy ^(^[^17^](#_ENREF_17)^)^ | Traffic light | ‘Green’ & ‘Amber’: ≥ 80% (‘Green’ aim 50% or more)  ‘Red’: < 20% | ‘Green’ options should be displayed in prominent areas.    ‘Red’ foods and drinks should not be displayed in excessive quantities or in prominent areas (e.g., checkout, entrance, waiting areas). | No supersizing or combos of any items unless they are ‘Green’.    ‘Green’ options should be sold at competitive prices where possible. | ‘Green’ options should be actively promoted.    ‘AMBER’ and ‘Red’ options should not be promoted (including any promotional material or activity). |
| Queensland, 2022 | A Better Choice Food and Drink Supply Strategy for Queensland Healthcare Facilities^(^[^18^](#_ENREF_18)^)^ | Traffic light | ‘Green’: ≥ 50% food and ≥50% drinks  ‘Amber’: artificially sweetened drinks <20% of drinks  ‘Red’: <20% food; ‘Red’ drinks should not be available | ‘Green’ drinks must make up at least 50% of drinks on display.    ‘Red’ foods must not make up more than 20% of all items on display.    Artificially sweetened drinks (classified as ‘Amber’) should not make up more than 20% of drinks on display.    Vending machines- ‘Green’ food must make at least 30% of all vending items on display. | ‘Red’ drinks should not be included in any price promotions. | ‘Green’ options should be actively promoted.    ‘Red’ drinks should not be promoted. |
| South Australia, 2023 | SA Health Policy: Healthy Food and Drink^(^[^19^](#_ENREF_19)^)^ / Food And Drink Classification Guide for South Australia - A System For Classifying Foods and Drinks 2023^(^[^20^](#_ENREF_20)^)^ | Traffic light | ‘Green’: ≥ 50%  ‘Amber’ diet drinks: < 20%  ‘Red’: < 20%, ‘Red’ drinks: < 10% | ‘Red’ foods must not make up more than 20% of all items on display. | N/A | ‘Green’ options  should be actively promoted and encouraged.    ‘Amber’ and ‘Red’ options should not be promoted |
| Victoria, 2020 | Healthy Choices: Policy Guidelines for Hospitals And Health Services, 2016 ^(^[^21^](#_ENREF_21)^)^ / Healthy Choices: Food and Drink Classification Guide, 2020 ^(^[^14^](#_ENREF_14)^)^ | Traffic light | ‘Green’: ≥ 50%, ‘Red’: < 20%  Policy directive^(^[^22^](#_ENREF_22)^)^: ‘Red’ drinks should not be available; ‘Amber’: artificially sweetened drinks <20% of drinks | ‘Green’ options should be displayed in prominent areas.    ‘Amber’ options should not be displayed but not at the expense of ‘Green’ options.    ‘Red’ should not be displayed in prominent areas. | ‘Red’ options should not be included in meal deals or point-of-sale promotions. | ‘Green’ foods should be promoted.    ‘Amber’ foods may be promoted, but not at the expense of ‘Green’ options.    ‘Red’ options should not be advertised or promoted. |
| Western Australia, 2022 | The Healthy Options WA: Food  and Nutrition Policy for WA Health Services and Facilities / Making Healthy Choices Easier-How to Classify Food and Drinks Guide ^(^[^23^](#_ENREF_23)^,^ [^24^](#_ENREF_24)^)^ | Traffic light | ‘Green’: ≥50%  ‘Amber’: < 50%; <25% intensely sweetened drinks  ‘Red’: <20% food; ‘Red’ drinks should not be available | ‘Green’ options must make up at least 50% of all items on display; ‘Red’ foods no more than 20% of all items on display.    Intensely sweetened drinks should not make up more than 25% of all drinks on display.    ‘Red’ and ‘Amber’ items must not be displayed in prominent locations (at eye level/arm’s length, near entrance and checkout etc.) | ‘Red’ and ‘Amber’ options must not be included in any price promotions. | Only ‘Green’ options can be promoted.    ‘Red’ and ‘Amber’ items must not be included in any marketing or promotional activity or material. |
| *The Star Choice™ Nutrient Criteria not publicly available. FOCIS Nutrient Criteria 2016^(^[^11^](#_ENREF_11)^)^ used for classification. | | | | | | |

**References**

1. ACT Government (2017) ACT Public School Food and Drink Policy. <https://www.education.act.gov.au/publications_and_policies/School-and-Corporate-Policies/school-administration-and-management/food-and-drink/act-public-school-food-and-drink-policy/act-public-school-food-and-drink-policy>] (accessed 5 Jun 2023)

2. Australian Government Department of Health (2013) National Healthy School Canteens, Guidelines for healthy foods and drinks supplied in school canteens. <https://www.health.gov.au/resources/publications/national-healthy-school-canteens-guidelines-for-healthy-foods-and-drinks-supplied-in-school-canteens>]

3. NSW Ministry of Health (2020) NSW healthy school canteen strategy food and drink criteria. <https://www.health.nsw.gov.au/heal/Publications/food-drink-criteria.pdf> (accessed 29 July 2021)

4. Northern Territory Government Department of Education (2017) School nutrition and healthy eating policy. <https://education.nt.gov.au/__data/assets/word_doc/0011/257807/policy_school_nutrition_and_healthy_eating.docx> (accessed 29 July 2021)

5. Queensland Health and Education Queensland (2020) Smart choices. Healthy food and drink supply strategy for Queensland schools, v1.4. <https://education.qld.gov.au/student/Documents/smart-choices-strategy.pdf> (accessed 5 June 2023)

6. Government of South Australia (2023) Food and Drink Classification Guide for South Australian Public Schools: A system for classifying foods and drinks 2023. <https://www.wellbeingsa.sa.gov.au/assets/downloads/Food-and-Drink-Classification-Guide-Public-Schools.pdf>

7. Tasmanian Government Department of Health (2021) School Food Matters, Smart Food Guide, A Whole School Approach to Eating Well. <https://www.schoolfoodmatters.org.au/wp-content/uploads/2021/12/SFM_Smart-Food-Guide_Dec-2021_P2.pdf>

8. State Government of Victoria (2012) Healthy Canteen Kit: School canteens and other school food services policy. <https://www.education.vic.gov.au/Documents/school/principals/management/gfylpolicy.pdf>

9. Government of Western Australia (2023) Student Health in Public Schools Procedures. <https://www.education.wa.edu.au/in/web/policies/-/student-health-care-in-public-schools-procedures> (accessed 10 September 2023)

10. Western Australian School Canteen Association Inc (WASCA) (2023) Star Choice™ Nutrient Criteria. <https://www.waschoolcanteens.org.au/star-choice-registration-program/star-choice-nutrient-criteria/>]

11. Federation of Canteents in Schools Inc (FOCiS) (2016) FOCiS Nutrient Criteria Review. <https://www.focis.com.au/wp-content/uploads/2017/05/FOCiS-Nutrient-Criteria-review-Summary-Report.pdf>] (accessed 10 September 2023

12. Northern Territory Government (2017) The healthy workplace toolkit. Your simple guide to workplace health and wellbeing. <https://digitallibrary.health.nt.gov.au/prodjspui/bitstream/10137/2725/1/Healthy%20Workplace%20Toolkit.pdf> (accessed July 30 2021)

13. Department of Health (2020) Healthy choices: healthy eating policy and catering guide for workplaces. <https://www2.health.vic.gov.au/public-health/preventive-health/nutrition/healthy-choices-for-retail-outlets-vending-machines-catering>

14. Department of Health (2020) Healthy Choices: food and drink classification guide. <https://www2.health.vic.gov.au/public-health/preventive-health/nutrition/healthy-choices-for-retail-outlets-vending-machines-catering>

15. ACT Health (2016) ACT Public Sector Healthy Food and Drink Choices Policy. <http://www.cmd.act.gov.au/__data/assets/pdf_file/0003/905772/WHS-01-2016-Healthy-Food-and-Drink-Choices-Policy.pdf> (accessed 4 Aug 2021)

16. NSW Ministry of Health (2017) Healthy food and drink in NSW health facilities for staff and visitors framework. <https://www.health.nsw.gov.au/heal/Publications/hfd-framework.pdf>. (accessed 29 July 2021)

17. Northern Territory Government (2017) Healthy choices made easy - healthy food and drink options for staff, volunteer and visitors, in NT health facilities. <https://digitallibrary.health.nt.gov.au/prodjspui/bitstream/10137/904/3/Healthy%20Food%20and%20Drink%20Options%20for%20Staff%2C%20Volunteers%20and%20Visitors%20in%20NT%20Health%20Facilities%20Policy.pdf> (accessed 29 July 2021)

18. Queensland Health (2022) A Better Choice Healthy Food and Drink Supply Strategy for Queensland Health Facilities. <https://hw.qld.gov.au/wp-content/uploads/2023/05/A-Better-Choice-Strategy-for-Healthcare.pdf>] (accessed 30 July 2023)

19. SA Health (2023) SA Health Policy: Healthy Food and Drink. <https://www.sahealth.sa.gov.au/wps/wcm/connect/b5ff2bc0-dda9-420e-a213-6e340d6f9be5/Healthy+Food+and+Drink+Policy_26+Jul+2023+v3.1.pdf?MOD=AJPERES&amp;CACHEID=ROOTWORKSPACE-b5ff2bc0-dda9-420e-a213-6e340d6f9be5-oN7HNcW> (accessed 30 August 2023)

20. Government of South Australia (2023) Food and Drink Classification Guide for South Australia - A system for classifying foods and drinks 2023. <https://www.preventivehealth.sa.gov.au/assets/downloads/Food-and-Drink-Classification-Guide.pdf> (accessed 30 August 2023)

21. Department of Health (2020) Healthy choices: food and drink guidelines for Victorian public hospitals. <https://www2.health.vic.gov.au/public-health/preventive-health/nutrition/healthy-choices-for-retail-outlets-vending-machines-catering>

22. Department of Health (2021) Healthy choices: policy directive for Victorian public health services. <https://content.health.vic.gov.au/sites/default/files/migrated/files/collections/policies-and-guidelines/h/healthy-choices-policy-directive-for-health-services.pdf>

23. Western Australia Department of Health (2022) Healthy Options WA Food and Nutrition Policy. <https://ww2.health.wa.gov.au/~/media/Corp/Policy-Frameworks/Public-Health/Healthy-Options-WA-Food-and-Nutrition-Policy/Healthy-Options-WA-Food-and-Nutrition-Policy.pdf> (accessed 4 Aug 2023)

24. Western Australia Department of Health (2022) Healthy Options WA: Making healthy choices easier-How to Classify Food and Drinks Guide. <https://ww2.health.wa.gov.au/~/media/Corp/Policy-Frameworks/Public-Health/Healthy-Options-WA-Food-and-Nutrition-Policy/Supporting/Making-Healthy-Choices-Easier-How-to-Classify-Food-and-Drink-Guide.pdf> (accessed 4 Aug 2023)
